# Research status and development trends of probiotics in clinical applications

**DOI:** 10.3389/fnut.2025.1650883

**Published:** 2025-09-09

**Authors:** Yang Li, Yucheng Yao, Zan Zhang, Yongqi Gan

**Affiliations:** ^1^School of Pharmacy, Guangxi Medical University, Nanning, China; ^2^Guangxi Institute for Drug Control, Nanning, China

**Keywords:** probiotics, clinical application, bibliometrics, visual analysis, Cite Space, VOS viewer

## Abstract

**Background:**

In recent years, the potential role of probiotics in modulating gut microbiota, enhancing immune function and improving metabolic diseases has become a highly field in biomedical and clinical research. The rapid increase in global probiotic clinical trials necessitates a systematic evaluation of current research status, developmental trends, and collaborative networks in this field.

**Purpose:**

This study employs bibliometric analysis to comprehensively evaluate research progress and hotspots in clinical applications of probiotics.

**Method:**

A systematic search was performed in the Web of Science database to collect articles and reviews regarding the clinical application of probiotics between 2000 and 2025. The retrieved records were analyzed using Microsoft Office Excel, Cite Space and VOS viewers.

**Result:**

During the period from 2000 to 2025, we retrieved a total of 3,674 papers related to the clinical application of probiotics, and the number of papers is showing a continuous growth trend. The research was mainly focused on North America, Western Europe, and East Asia, with the United States leading the way with 714 papers, high impact (H-index 107, total citations 44,833), and top institutions (Harvard Univ). Nutrition and microbiology are the main academic fields. The current research hotspots and development directions focus on the application of probiotics in diseases such as “inflammation”, “obesity”, “insulin resistance”, “depression”, “hyperlipidemia”, and “cancer”.

**Conclusion:**

This study represents the first application of bibliometric methods to systematically visualize and analyze research progress in the field of probiotic clinical applications, identifying key research trends and frontiers. The findings offer valuable insights for researchers at various career stages, particularly those new to the field, enabling them to identify critical developments and serving as a foundational reference for future clinical applications of probiotics.

## 1 Introduction

In 1907, Russian scientist Elie Metchnikoff first proposed the concept of probiotics. His observations of Bulgarian farmers' longevity associated with long-term consumption of fermented dairy products led him to hypothesize that lactic acid bacteria conferred health benefits. Metchnikoff proposed that these bacteria could inhibit pathogenic intestinal microbiota, potentially extending lifespan. This theory established the scientific foundation for probiotic research. In 1965, Lilly and Stillwell first coined the term “Probiotics”, originally defining them as “microbial-derived substances that stimulate the growth of other probiotics.” Over time, this definition has evolved to its current form: “live probiotics that confer health benefits when administered in adequate amounts”. Advances in molecular biology and microbiological techniques have enabled researchers to elucidate probiotic mechanisms of action, including microbiota modulation, immune function enhancement, and prevention effects (1–3). This scientific progress initiated a new era of probiotic research.

Probiotics are live microorganisms defined by the Food and Agriculture Organization of the World (FAO) and the World Health Organization (WHO). Recent advances have led to the conceptualization of “next-generation probiotics (NGP)”, which were defined as living biological therapeutic drugs (4). As functional microorganism with significant potential, NGPs demonstrate broad application prospects across multiple domains including food science, medical therapeutics, and health management. Probiotics are mainly classified into the following categories: *Lactobacillus*, (such as *Lactobacillus acidophilus, Lactobacillus rhamnoses*, and *Lactobacillus plantarum*, etc.), *Bifidobacterium*, (such as *Bifidobacterium infants, Bifidobacterium longum, Bifidobacterium breve*, etc.), *Yeast*, (such as S*accharomyces bouvardia*), Other bacterial genera, (such as *certain streptococcus* (including *Streptococcus thermophilus*) and *Bacillus* (including *Bacillus coagulase*) etc. Probiotics have been extensively applied in clinical practice, demonstrating efficacy in disease prevention, treatment and adjunctive management. They exhibit significant therapeutic value across multiple medical domains, particularly in gastrointestinal disorders, immune-mediated conditions, metabolic diseases, women's health, and mental health (5). Recent years have witnessed exponential growth in publications concerning probiotic clinical applications. As research advances, the mechanisms of probiotics action are being elucidated with increasing comprehensiveness, while personalized therapies and novel probiotic formulations are emerging as key research priorities. This bibliometric analysis delineates the developmental trajectory of probiotic research through visual analytics. Employing a chronological framework, we systematically analyze research hotspots, identify emerging trends, and critically evaluate both major advances and persistent challenges in probiotic clinical applications over the past three decades. Consequently, this review provides valuable insights to guide future probiotic development and facilitate innovation in therapeutic translation.

## 2 Materials and methods

### 2.1 Data source and search strategy

The study retrieved bibliographic data from the Web of Science, spanning publications from 2000 to February 23, 2025. This analysis focused on identifying research hotpots and developmental trends in probiotic research. The core database was used as the basic data for the research. The reason why Web of Science was selected as the primary data source due to its comprehensive coverage of over 12,000 high-impact journals indexed in the Science Citation Index (SCI), Social Sciences Citation Index (SSCI), Arts & Humanities Citation Index (A&HCI). Compared to alternative databases such as Scopus, Medline and PubMed, Web of Science offers superior bibliometric analysis capabilities, as evidenced by its widespread adoption in visualization studies and established academic authority. The search strategy employed the title field query: [TI = (Probiotic^*^) AND TI = (“Clinical practice^*^” OR “clinical using” OR “clinical use” OR “clinical application” OR “clinical study” OR “clinical studies” OR “clinical trial^*^” OR “clinical usage” OR “clinical utility”)] OR [AB = (Probiotic^*^) AND AB = (“Clinical practice^*^” OR “clinical using” OR “clinical use” OR “clinical application” OR “clinical study” OR “clinical studies” OR “clinical trial^*^” OR “clinical usage” OR “clinical utility”)] OR [AK = (Probiotic^*^) AND AK = (“Clinical practice^*^” OR “clinical using” OR “clinical use” OR “clinical application” OR “clinical study” OR “clinical studies” OR “clinical trial^*^” OR “clinical usage” OR “clinical utility”)] search queries.

### 2.2 Data processing

(I) Inclusion Criteria.

(1) Literature related to the clinical application of probiotics; (2) Literature published in English; (3) Literature types include origin, art ide and review; (4) The literature information must be complete (including title, country, author, keywords, source, etc.).

(II) Exclusion Criteria.

(1) Duplicate publications (2) Self-cited publications.

### 2.3 Data normalization

The selected records were exported in plain text format with all special characters removed. Subsequently, duplicate publications were identified and excluded using the Cite Space software.

### 2.4 Methods and data analysis

This study employed bibliometric methods to analyze research hotpots, advances and frontiers in the field. The bibliometric analysis was conducted using RStudio, VOS viewer, Cite Space and Microsoft Excel with the bib-biomatrix package. Among these tools, VOS viewer and Cite Space have gained widespread recognition in academic circle (6). Specifically, VOS viewer is a bibliometric visualization tool developed by van Eck and Waltman at Leiden University's Center for Science and Technology Studies. In addition, the VOS viewer demonstrates distinct advantages in bibliometric analyses including keyword co-occurrence mapping, cluster identification, and research hotspot visualization (7). Cite Space was proposed by Professor Chen at Drexel University, it is a special bibliometric analysis tool designed for visualizing temporal patterns and emerging trends in scientific literature through citation network analysis and cluster mapping (8, 9). Visual and serialized knowledge maps were used to display the complex relationships among keywords, authors, institutions, countries and other information elements, including interactions, intersections and evolutions. The Cite Space software focuses on expressing relationship strength between various themes through tree diagrams and connecting lines, while VOS viewer software primarily illustrates clustering relationships among structural nodes through distance and density visualization. The advantages of Cite Space and VOS viewer software can complement each other (10). The implementation and development of these two-software tool have significantly advanced research progress and application expansion in the field of information visualization. Accordingly, this study employed VOS viewer to analyze co-occurrence knowledge maps of authors, institutions, and countries in probiotic research, and utilized Cite Space to examine co-citation bursts in the literature.

## 3 Result

### 3.1 Literature retrieval and screening results

**Figure 1:**
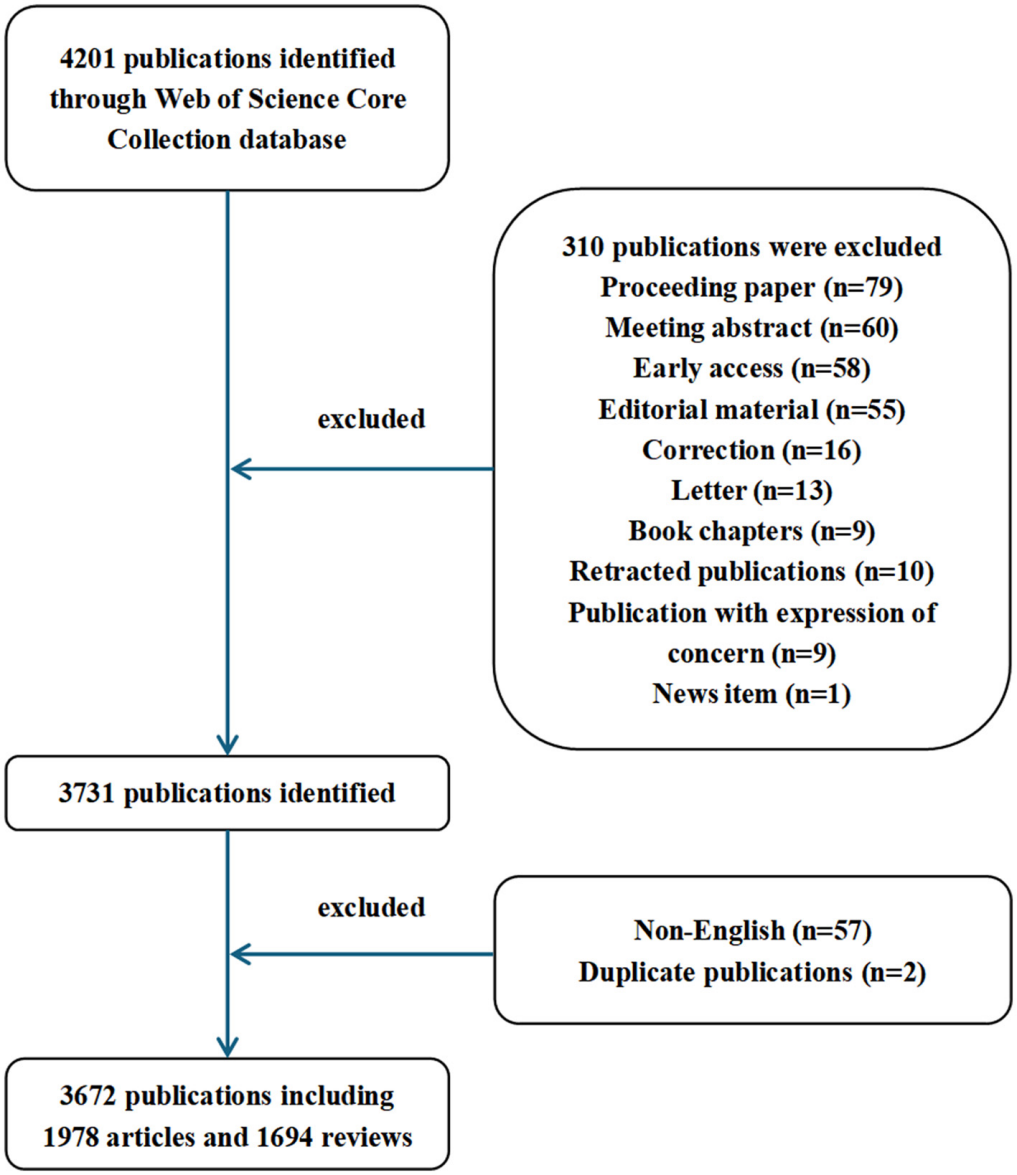
Flowchart for the data selection process.

We initially searched 3,674 documents in the WOS database. After applying the predefined inclusion and exclusion criteria, 3,672 publications were included for analysis. The specific steps are shown in Figure 1.

### 3.2 Analysis of the annual number of publications and citations

**Figure 2:**
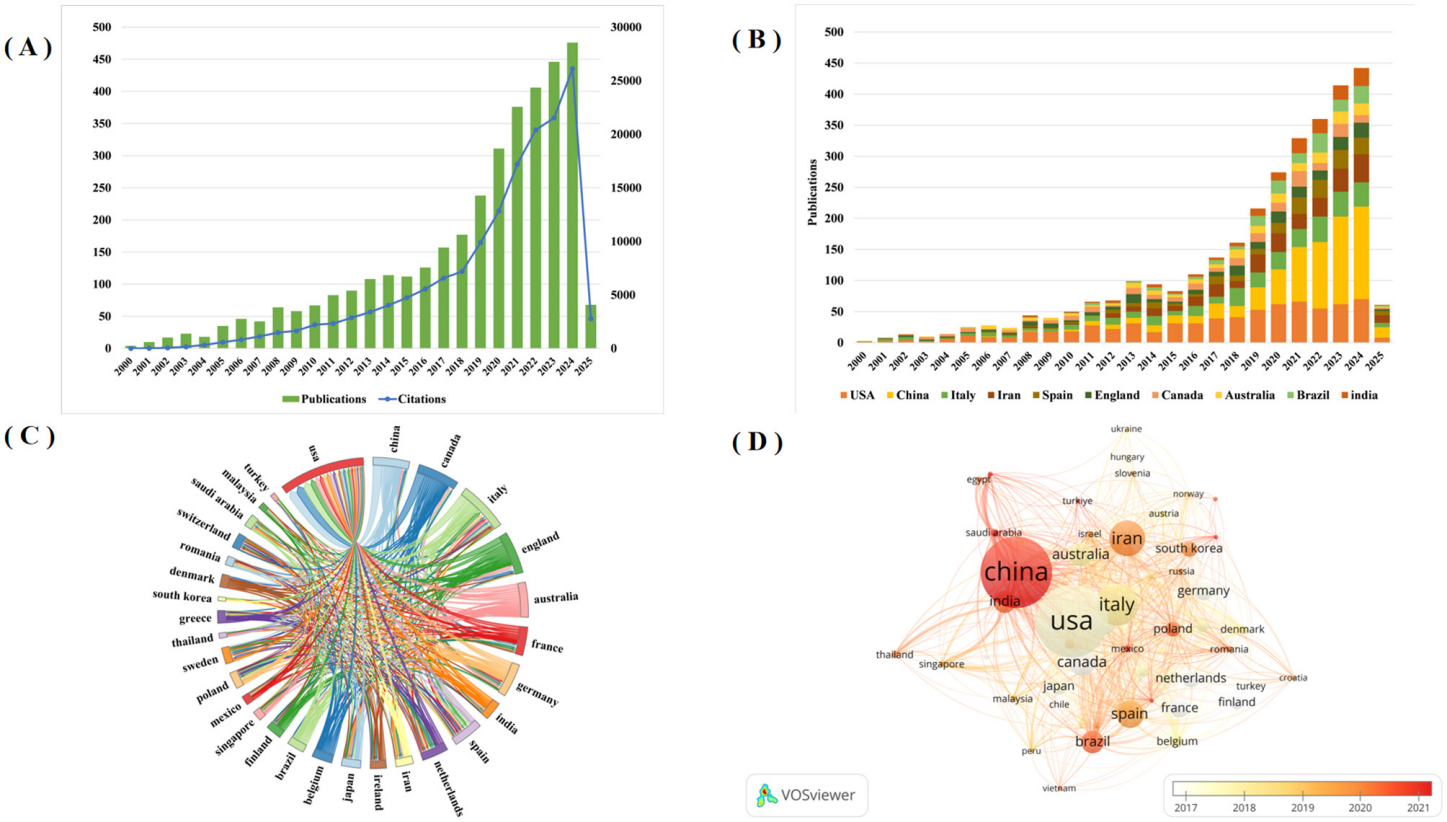
**(A)** The global trend in the annual number of Probiotics in Clinical Applications publications and citation from 2000 to 2025; **(B)** The annual number of publications in the top 10 most productive countries; **(C)** Scientific cooperation between the top 30 countries in terms of the number of publications worldwide; **(D)** Overlay visualization map of country co-authorship analysis generated by VOS viewer software.

Publication volume within a given timeframe serves as a reliable indicator of research activity and developmental trends in a field. Figure 2A shows the global trends from 2000 to 2025. The analysis reveals a consistent upward trajectory in probiotic clinical application research. Annual publications first surpassed 100 in 2013, demonstrated accelerated growth since 2019, and peaked in 2024 with 476 articles. These findings demonstrate growing research interest in this field, suggesting continued increases in publication output. As of February 2025, the dataset comprised 3,672 publications, which collectively received 155,819 citations (excluding 142,772 self-citations).

### 3.3 Analysis of the number of publications and cooperation among countries

The study identified 99 countries that have contributed publications in this research domain. Table 1 shows the top ten productive countries are ranked by publication volume. The United States leads with the highest publications count (714), H-index (107), and total citations (44,833). China and Italy follow in second and third positions with 699 and 355 publications, respectively. These findings demonstrate particularly strong research engagement in probiotic clinical applications from these three nations. For Average number of citations, Canada ranked first in average citation count (83.26), followed by the United Kingdom (78.37) and the United States (62.79). Furthermore, Figure 2B shows the annual publication growth among the top ten most productive countries. Notably, while the United States maintained research dominance prior to 2021, China's annual publication output has exceeded that of the United States since 2021, demonstrating a rapid growth trajectory.

**Table 1:** Top 10 countries in terms of number of publications.

**Rank**	**Countries**	**Counts**	**H-index**	**TC**	**ACI**
1	USA	714	107	44,833	62.79
2	China	699	69	19,160	27.41
3	Italy	355	67	14,550	40.99
4	Iran	293	58	11,589	39.55
5	Spain	209	50	9,436	45.15
6	England	204	54	15,988	78.37
7	Canada	199	60	16,568	83.26
8	Australia	174	52	9,336	53.66
9	Brazil	166	35	5,157	31.07
10	India	160	35	5,041	31.51

ACI, average citations per item; TC, total citations.

Figure 2C shows the collaboration networks among the top 30 most productive countries. The United States demonstrated the strongest international collaborations, with China (59), Canada (57), Italy (52), England (36) and Australia (31). In addition, we also used the VOS viewer software to create a temporal co-authorship network among countries, as shown in Figure 2D, where node size corresponds to publication volume and color gradient (white-orange-dark red) indicates the average publication year. This visualization reveals that Canada, France, and the Netherlands were early contributors in this field, while China emerged more recently.

### 3.4 Analysis of the number of publications and collaborations of key authors

**Figure 3:**
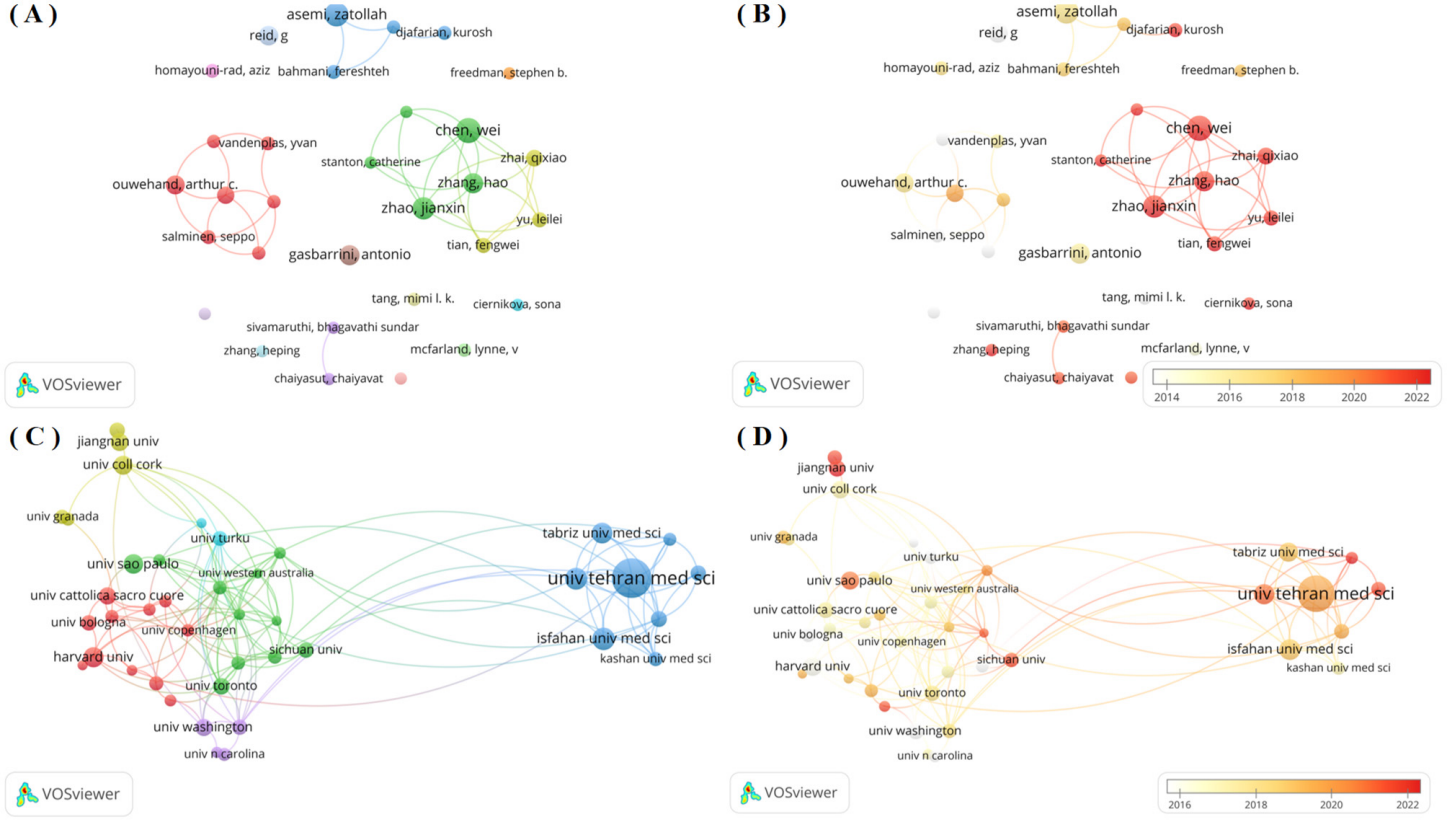
**(A)** Network visualization map of author co-authorship analysis generated by VOS viewer software; **(B)** Overlay visualization map of author co-authorship analysis generated by VOS viewer software; **(C)** Network visualization map of institution co-authorship analysis generated by VOS viewer software; **(D)** Overlay visualization map of institution co-authorship analysis generated by VOS viewer software;

Table 2 shows the top ten authors probiotic clinical application research, ranked by publication output. Chen, Wei ranked first with 27 publications, followed by Asemi Zatollah and Zhao jianxin with 25 and 23 publications. Asemi Zatollah achieved the highest H-index of 22 and total citations of 2,636, while Reid G ranked second with an H-index of 16 and total citations of 2,633. Among these ten authors, six were affiliated with Chinese institutions, with the remaining four representing Iran, Italy, Canada and Finland respectively. Figure 3A shows the author collaboration network. The network graph shows that there are at least 10 published papers with a total of 31 authors. Authors are clustered by node color, enabling intuitive identification of core researchers and collaborative teams. Analysis revealed three core research groups: (1) the red cluster (Ouwehand AC, Lebeer S, et al.), (2) the green cluster (Chen W, et al.), and (3) the blue cluster (Asemi Z, et al.). These groups represent the dominant research teams in this field. Figure 3B shows the time overlap graph of cooperation among authors, revealing that Chinese researchers including Chao Wei, Zhao hao and Zhai qixiao are represent more recent contributors in this field.

**Table 2:** Top 10 authors in terms of number of publications.

**Rank**	**Authors**	**Counts**	**H-index**	**TC**	**ACI**	**Institutions and country**
1	Chen, Wei	27	13	656	24.30	Jiangnan University and China
2	Asemi, zatollah	25	22	2,636	105.44	Kashan University and Iran
3	Zhao, jianxin	23	12	627	27.26	Jiangnan University and China
4	Zhang, hao	20	5	112	5.60	Jiangnan University and China
4	Gasbarrini, antonio	20	12	542	27.10	Sacred Heart Catholic University and Italy
5	Reid, g	19	16	2,633	138.58	Western Ontario University and Canada
6	Ouwehand, arthurc	18	14	994	55.22	International Flavors & Fragrances Inc and Finland
7	Zhai, qixiao	16	10	295	18.44	Jiangnan University and China
8	Tian, fengwei	15	9	408	27.20	Jiangnan University and China
9	Yu, leilei	13	8	194	14.92	Jiangnan University and China

ACI, average citations per item; TC, total citations.

### 3.5 Analysis of literature publishing institutions

Institutional analysis identified approximately 12,610 research-active institutions in this field. Table 3 shows the top 10 institutions in terms of the number of publications. Among the analyzed institutions, Univ Tehran Med Sci ranks first with 101 publications, significantly surpassing other institutions. Isfahan Univ Med Sci and Shahid Beheshti Univ Med Sci followed with 52 and 51 publications respectively. Notably, Harvard Univ from the United States achieved the highest H-index (34) and total citations (5,030). Institutional collaboration networks reflect both academic influence and research cooperation patterns (11). Accordingly, we employed VOS viewer software to conduct a network graph analysis of the cooperation among research institutions that have published at least 20 papers, as shown in Figure 3C. The network comprises 41 institutions clustered into 6 distinct groups, revealing strong collaborative relationships between Univ Tehran Med Sci, Monash Univ and Univ Melbourne of Melbourne and their partners. However, several institutions including Univ Granada, Jiangnan Univ, exhibited more dispersed nodal connections with limited internal cohesion and variable research output. These patterns suggest comparatively lower publication volumes, reduced academic influence, and less developed collaborative networks among these institutions, indicating an absence of large-scale institutional research partnerships. Additionally, we have also produced a temporal overlap chart of inter-agency cooperation as shown in Figure 3D, which identifies more recent contributors to this field, including Shiraz Univ and Jiangnan Univ (represented by dark red nodes).

**Table 3:** Top 10 institutions in terms of number of publications.

**Rank**	**Institution**	**Counts**	**Country**	**H-index**	**TC**	**ACI**
1	Univ Tehran Med Sci	101	Iran	32	4,121	40.80
2	Isfahan Univ Med Sci	52	Iran	25	2,189	42.10
3	Shahid Beheshti Univ Med Sci	51	Iran	21	1,334	26.16
4	Tabriz Univ Med Sci	47	Iran	25	2,481	52.79
5	Harvard Univ	46	USA	34	5,030	109.35
6	Univ Sao Paulo	45	Brazil	17	1,585	35.22
7	Univ Coll Cork	42	Ireland	29	3,917	93.26
8	Jiangnan Univ	40	China	18	1,084	27.10
9	Univ Cattolica Sacro Core	38	Italy	21	1,487	39.13
9	Univ Washington	38	USA	13	808	21.26

ACI, average citations per item; TC, total citations.

### 3.6 Analysis of the most influential journals

For centuries, peer-reviewed scientific publications have served as a fundamental medium for interdisciplinary scholarly communication. Publication in international journals remains essential for establishing valid scientific discourse and disseminating research findings (12, 13). Analysis of journal source distribution enables researchers to efficiently identify the most suitable publication venues for their work. Our findings indicate that publications on probiotic clinical applications are disseminated across approximately 1,000 distinct journals. Table 4 summarizes the basic information of the top 10 journals with the largest number of publications in this field. Among them, *NUTRIENTS* (201) produced the most, followed by *MICROORGANISMS* (77) and *FRONTIERS IN MICROBIOLOGY* (63). A journal's Journal Impact Factor (JIF) serves as a key parameter for assessing both the journal's academic influence and the citation impact of its published articles, representing a 2-year moving average of citation frequency (14). Among the top 10 academic journals, *CLINICAL NUTRITION* (6.6) had the highest JIF, followed by *INTERNATIONAL JOURNAL OF MOLECULAR SCIENCES* (4.9) and *NUTRIENTS* (4.8), etc. Accordingly, the journal citation reports also divide the journals belonging to the same discipline category in the Web of Science database into four equal parts based on the JIF value. The top 25% are attributed to Q1, the top 25-50% are attributed to Q2, and so on. It can be seen from Table 4 that 4 journals belong to Q1, 4 journals belong to Q2, and 2 journals belong to Q1/Q2. In addition, geographical distribution analysis revealed that five of the ten journals originate from Switzerland, three from the United States, with the remaining two based in the United Kingdom and the Netherlands respectively. Notably, these high-activity journals are located in Western Europe and North America. The absence of East Asian representation, particularly Chinese journals, in this ranking suggests the need for enhanced international journal development in Asian countries to strengthen their academic influence. It is noteworthy that the Chinese government has implemented substantial investments and policy incentives to foster international journal development in recent years (15).

**Table 4:** Top 10 journals in terms of citations.

**Rank**	**Journal**	**Counts**	**TC**	**H-index**	**Country**	**JIF (2024)**	**JCR (2024)**
1	NUTRIENTS	201	6,241	45	Switzerland	4.8	Q1
2	MICROORGANISMS	77	1,249	20	Switzerland	4.1	Q2
3	FRONTIERS IN MICROBIOLOGY	63	2,117	24	Switzerland	4.0	Q2
4	BENEFICIAL MICROBES	59	1,587	25	Netherlands	3.0	Q2
5	INTERNATIONAL JOURNAL OF MOLECULAR SCIENCES	55	2,026	25	Switzerland	4.9	Q1/Q2
6	FRONTIERS IN NUTRITION	51	509	13	Switzerland	4	Q2
7	PLOS ONE	46	1,969	25	USA	2.9	Q1
8	CLINICAL NUTRITION	42	2,937	26	England	6.6	Q1
8	PROBIOTICS AND ANTIMICROBIAL PROTEINS	42	990	19	USA	4.4	Q1/Q2
9	WORLD JOURNAL OF GASTROENTEROLOGY	41	3,654	29	USA	4.3	Q1

TC, total citations.

### 3.7 Analysis of the most relevant topic categories

**Figure 4:**
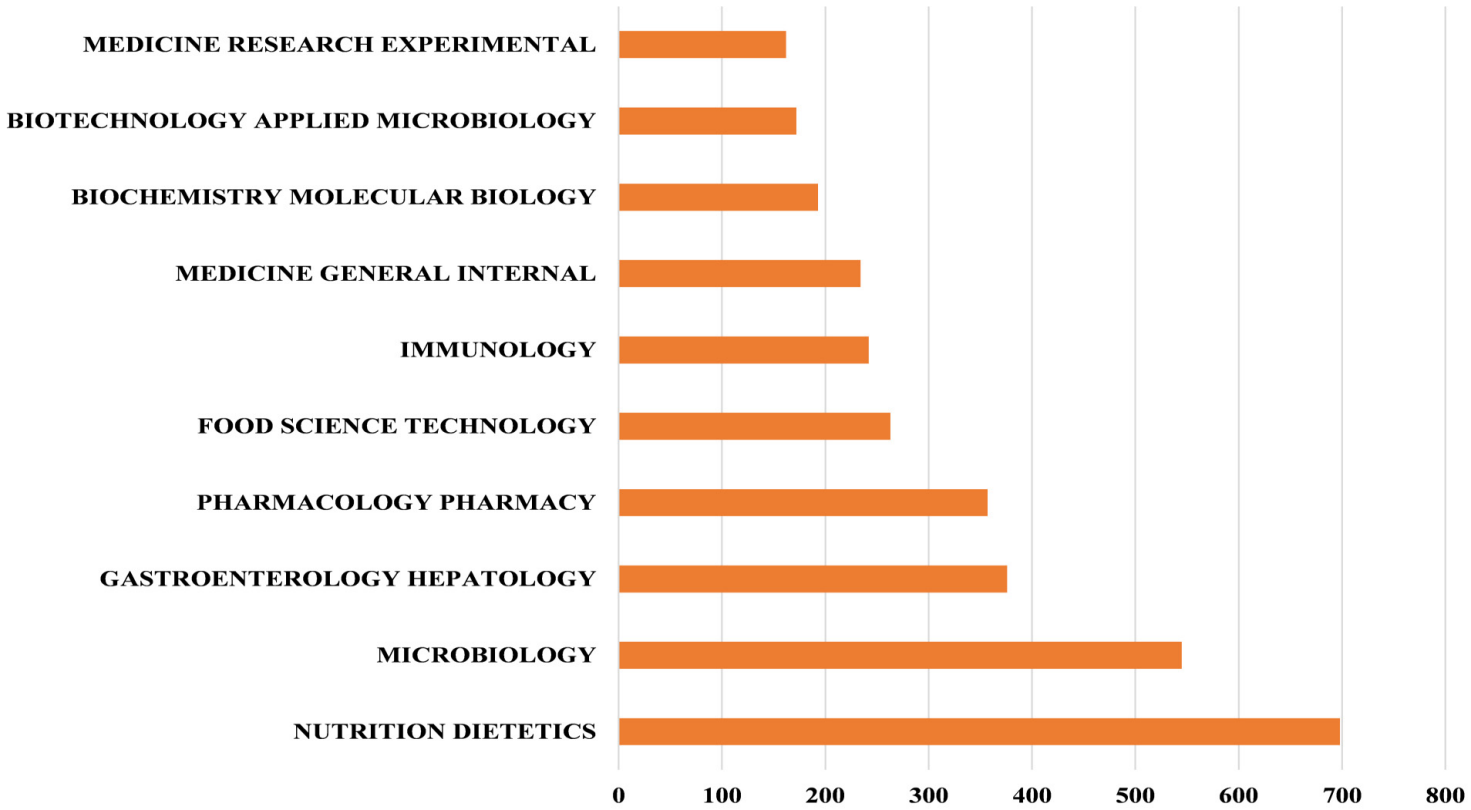
The top 10 subject categories with the highest number of publications.

**Figure 5:**
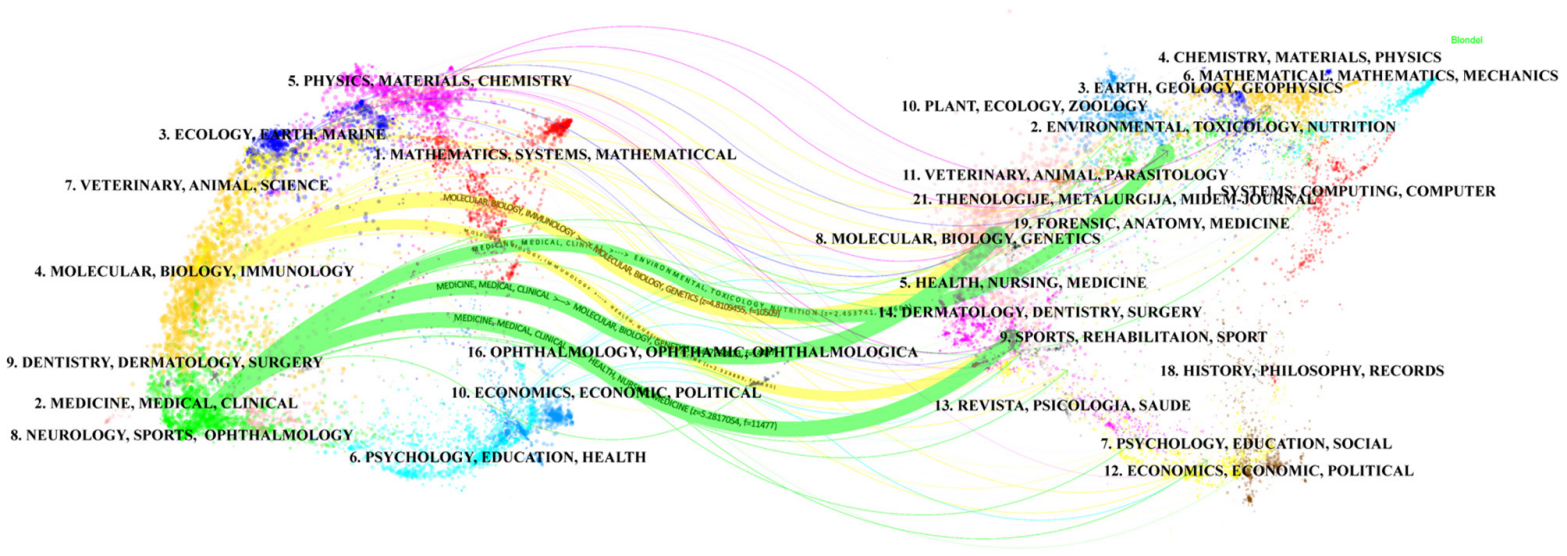
The dual-map overlay of academic journals generated by Cite Space software.

In the Web of Science database, each publication is assigned one or more subject categories for efficient retrieval. Figure 4 shows the top 10 subject categories with the highest number of publications. We found that NUTRITION, DIETETICS, MICROBIOLOGY and GASTROENTEROLOGY HEPATOLOGY are the most prominent and frequently represented subject categories in this research domain. Furthermore, the dual-map overlay visualization delineates the disciplinary distribution of journals publishing probiotic clinical application research, as shown in Figure 5. The dual-map overlay is generated from 10,000 source journals indexed in Web of Science. Using this dataset, citation trajectories were established through the dual-map overlay module. This visualization method clearly demonstrates interdisciplinary knowledge flow and identifies disciplinary research hotpots, with citing journals displayed on the left and cited journals on the right. The colored paths indicate the citation relationships. On the figure, we can see the following five core reference paths. Two orange paths said published in Molecular Biology/Immunology journal literature usually references published in Molecular Biology/Genetics and Health/Nursing/Medicine journal literature. The green path means that most papers published in medical/medical/clinical journals may tend to cite those published in environmental/toxicology/nutrition and molecular/biological/genetic as well as health/nursing/medical journals.

### 3.8 Analysis of highly cited literature

The Web of Science enables multiple classification approaches for scholarly literature, allowing identification of articles that have garnered significant attention within the scientific community. Consequently, citation analysis serves as a principal methodology in bibliometric investigations. While some debate persists regarding the interpretation of citation metrics (16), the academic consensus maintains that citation frequency correlates with a publication's scholarly influence, wherein higher citation counts typically reflect greater academic impact within a given field (17). Table 5 lists the top 10 most frequently cited papers in clinical application research related to probiotics. For each article, we have provided the journal name, citation count, paper title, first author's name and publication year. The journals presented in this table are ranked by publication volume. Specifically, No. 1 is the “Gut-Brain axis:” by Foster, JA, published IN *TRENDS IN NEUROSCIENCES* the article “how the microbiome influences anxiety and depression” has been cited 1,572 times and is the most frequently cited paper in this field. The research results of this article indicate that the bacteria in the gastrointestinal tract (GI), including symbiotic bacteria, probiotics and pathogenic bacteria, can activate neural pathways and the central nervous system (CNS) signaling system, further demonstrating that the microbiota plays a very important role in normal brain functions. This provides new approaches for understanding ongoing or future animal clinical studies on the microbiota-gut-brain axis in the prevention and treatment of mental illnesses, including anxiety and depression. The second most highly cited paper is “Precision microbiome reconstitution restores bile acid mediated resistance to Clostridium difficile.” published in *NATURE* by Buffie, CG et al, this article has been cited 1,294 times (18), It is indicated that when antibiotics disrupt the microbiota, the risk of infection increases. However, specific intestinal bacteria (such as Clostridium glitter) enhance their resistance to Clostridium difficile infection through bile acid metabolism, thereby reducing antibiotic-induced diarrhea. The third most frequently cited paper is “The Role of Probiotics and Prebiotics in Gut Health and Diseases: From Biology to Clinical Practice” published by Sanders, ME et al.in *NATURE REVIEWS GASTROENTEROLOGY & HEPATOLOGY*. In this review, insights into the mechanisms by which probiotics and prebiotics affect health are discussed, and facts and supporting information regarding their clinical application and use are reaffirmed, as well as prospects for the future are presented (19).

**Table 5:** Top 10 references with citations related to clinical applications of probiotics.

**Rank**	**Title**	**Frist author**	**Citations**	**Journal**
1	Gut-brain: how the microbiome influences anxiety and depression	Foster, JA	1,572	TRENDS IN NEUROSCIENCES
2	Precision microbiome reconstitution restores bile acid mediated resistance to Clostridium difficile	Buffie, CG	1,294	NATURE
3	Probiotics and prebiotics in intestinal health and disease: from biology to the clinic	Sanders, ME	1,105	NATURE REVIEWS GASTROENTEROLOGY & HEPATOLOGY
4	Role of gut microbiota in type 2 diabetes pathophysiology	Gurung, M	1,023	EBIOMEDICINE
5	The International Scientific Association of Probiotics and Prebiotics (lSAPP) consensus statement on the definition and scope of postbiotics	Salminen, S	1,011	NATURE REVIEWS GASTROENTEROLOGY & HEPATOLOGY
6	Assessment of psychotropic-like properties of a probiotic formulation (Lactobacillus helveticus Ro052 and Bifidobacterium longum RO175) in rats and human subjects	Messaoudi, M	964	BRITISH JOURNAL OF NUTRITION
7	Canadian clinical practice guidelines for nutrition support in mechanically ventilated, critically ill adult patients	Heyland, DK	957	JOURNAL OF PARENTERAL AND ENTERAL NUTRITION
8	European Society of Clinical Microbiology and Infectious Diseases: update of thetreatment guidance document for Clostridium difficile infection	Debast, SB	882	CLINICAL MICROBIOLOGY AND INFECTION
9	Health benefits of fermented foods: microbiota and beyond	Marco, ML	835	CURRENT OPINION IN BIOTECHNOLOGY
10	Ulcerative colitis	Farrell, RJ	822	LANCT

### 3.9 Reference literature explosion application

**Figure 6:**
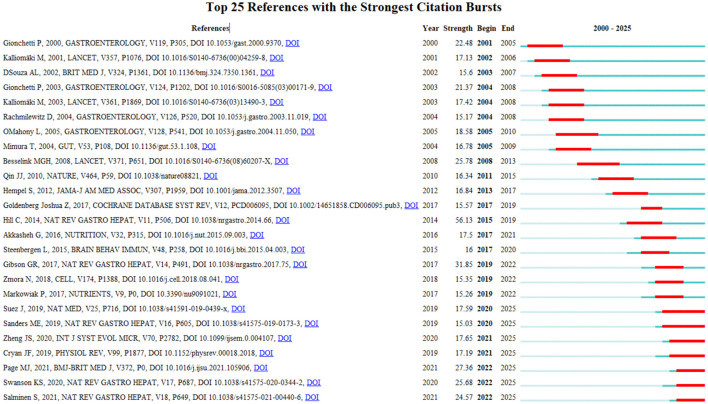
Visualization map of top 25 references with the strongest citation bursts by Cite space software.

Research frontiers represent the most promising and potentially research impactful directions in scientific investigation. Kleinberg's burst detection algorithm serves as an effective analytical tool for identifying sudden increases in citation frequency or keyword usage within defined time periods. This method provides an effective way to identify the concepts and track evolving trends in probiotic clinical research. The Burst Detection function in Cite Space identifies keywords demonstrating significant citation surges within defined time periods, with these results serving as valuable indicators for research frontier analysis (20). This study employed Cite Space's burst detection function to extract emerging keywords related to probiotic clinical applications. Figure 6 shows the top 25 references with the strongest citation bursts. In this picture, the blue line represents the time interval, and the red part represents the time period during which the reference outbreak occurred. Among these references, the one with the strongest burst value was written by Gionchetti et al. (21), their research revealed that probiotic preparations could significantly reduce postoperative chronic risk in ulcerative colitis patients following ileal pouch-anal anastomosis. In addition, the second most prominent citation burst was observed for the publication by Kalliomäki et al. (22), which outbreak lasted for 4 years. The article details how oral administration of lactic acid bacteria in children with specific diseases can enhance endogenous TGF-β and IL-10 in the body, Moreover, these anti-inflammatory cytokines play a key role in the prevention and treatment of atopic disease, perhaps even more important than the role of T helper type 1 inducible factor. Notably, while citation bursts have subsided for most references, several persist, suggesting sustained research interest in these topics in recent years.

### 3.10 Key word co-occurrence analysis

**Figure 7:**
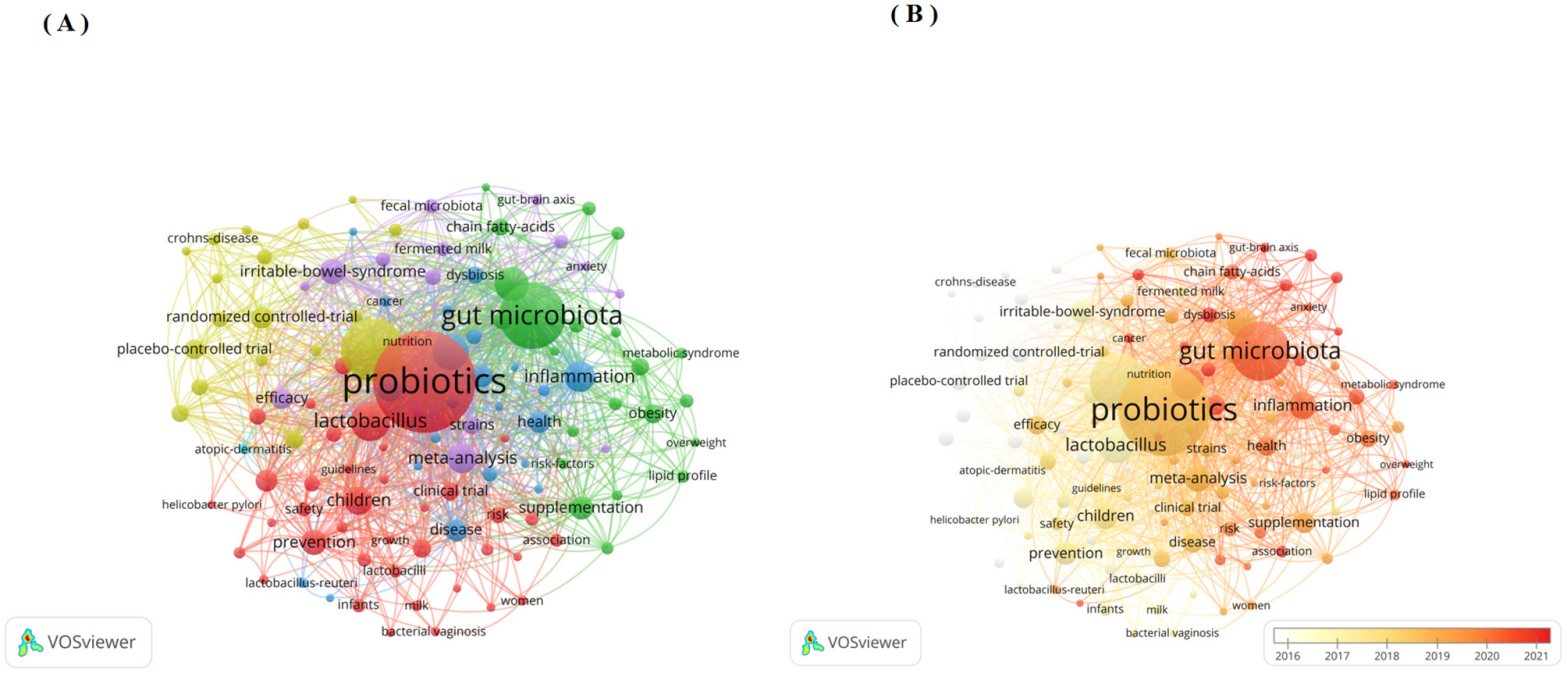
**(A)** Network visualization map of authors' keywords co-occurrence analysis by VOS viewer software; **(B)** Overlay visualization map of authors' keywords co-occurrence analysis by VOS viewer software.

Beyond cited references, keywords equally represent the central themes and core concepts of a research topic (23). Keyword co-occurrence analysis represents another established bibliometric method for detecting research hotspots. This approach determines keyword relevance through their joint appearance frequency in publications (13). This study employed VOS viewer software to perform keyword co-occurrence analysis. We established a threshold to a minimum co-occurrence of 50 times, and after merging synonyms and removing meaningless keywords, there are total of 118 keywords as shown in Figures 7A, B. Node size corresponds to keyword occurrence frequency, while inter-node distance inversely reflects relationship strength. Line thickness indicates co-occurrence frequency between terms (13). Figure 7A demonstrates 6 keyword clusters, the largest cluster is the red cluster, with 39 keywords. Furthermore, Figure 7B identifies current research hotspots through deep-red nodes, including “dysbiosis”, “fecal microbiota transplantation” and “gut-brain axis”. Furthermore, we have listed the top 20 keywords related to clinical applications as shown in Table 6.

**Table 6:** The top 20 keywords related to clinical applications.

**Rank**	**Keywords**	**Occurrence frequency**
1	Inflammation	357
2	Lactobacillus-rhamnosus gg	214
3	Obesity	172
4	Infection	135
5	Ulcerative-colitis	121
6	Insulin-resistance	108
7	Depression	106
8	Crohns-disease	93
9	Lipid profile	91
10	Inflammatory-bowel-disease	87
11	Necrotizing enterocolitis	87
12	Anxiety	78
13	Cancer	76
14	Atopic-dermatitis	71
15	Ulcerative colitis	64
16	Bacterial vaginosis	61
17	Overweight	56
18	Sepsis	55
19	Colorectal-cancer	53
20	Periodontitis	51

## 4 Discussion

### 4.1 Basic information

This study employed Cite Space and VOS viewer for quantitative analysis of probiotics clinical applications, systematically summarizing research findings and advancements. Quantitative analysis of journals by publication volume, country, authors, institutions, subject, periodicals and other basic information. Analysis of publication volume reveals 3,604 articles on probiotic clinical applications since 2000, demonstrating a consistent growth trend. Higher citation frequency correlates with greater scientific influence and quality within this research domain. As shown in Figure 2, citation frequency in this field demonstrates a consistent annual increase. Based on the statistical analysis of publication outputs across countries/regions and institutions, key countries/regions and research institutions in probiotic clinical applications, while simultaneously mapping their collaborative networks. The United States, China and Italy are the main countries for researching the clinical applications related to probiotics. Research on the clinical application of probiotics in countries such as the United States, China and Canada is relatively mature. Among the top ten institutions, four are from Iran, two are from the United States, one is from Brazil and one is from Italy. Notably, China represents only one entry. Among the included institutions, Univ Tehran Med Sci ranked first in both publication output and H-index. The results demonstrate close international and interinstitutional collaboration. Such cooperative networks facilitate the elimination of academic barriers and promote advancements in probiotic clinical research. Among the top 10 authors, Chen, Wei (24) who published the most articles, followed by Asemi, zatollah (25) and Zhao, jianxin (23). It indicates that these three authors have made the most outstanding contributions in the field of clinical application research related to probiotics. Professor Asemi zatollah from Kashan University of Iran is the author with the highest H-index, which is a comprehensive quantitative indicator used to evaluate the quantity and level of academic output of researchers (26). Professor Asemi, zatollah demonstrated that probiotic supplementation may significantly improve wound healing in diabetic foot ulcers, clinical symptoms in patients with polycystic ovary syndrome, metabolic indicators in patients with diabetic retinopathy, and clinical characteristics of nervous system metabolism through randomized, double-blind, and placebo-controlled trials (24, 25, 27), Especially, it has a significant effect on the clinical, metabolic and genetic status of Alzheimer's disease (24). The second ranking institution is the Western Ontario University of Canada Reid, g. He believes that Bifidobacterium not merely as an evolutionary feature of the maternal vagina and differentiating microbiota, capable of regulating immune responses to produce short-chain fatty acids, enhancing the intestinal barrier, and becoming a core component for the healthy growth of infants (28), but also can acidify the local environment and prevent the colonization of urological and gynecological pathogens, reducing the risks of bacterial vaginosis, urinary tract infections, cystitis, and chronic kidney disease (29, 30). Third on the list is Ouwehand arthur c from International Flavors and Fragrances Inc of Finland, which found that the saccharide*2*′*-fucosyl lactose2*′*-FL* and *Bifidobacterium infantile* subspecies (*B. infantis*), these microorganisms demonstrate potential symbiosis in infant immune system development, significantly influencing immunological maturation (31). The study further elucidates the mechanistic pathway through which the novel prebiotic combination—chitin glucan (CG) with *Lactobacillus acidophilus* NCFM—attenuates visceral nociception and intestinal inflammation, while also delineating probiotic-mediated microbiota modulation as a critical therapeutic strategy (32–34). Notably, he realized the microbiota in mood disorders-im-brain axis from preclinical to the transformation of the clinical research challenges (35). Analysis of literature source distribution facilitates identification of core journals publishing probiotic clinical research and supports researchers in establishing scientific contributions. These findings demonstrate significant interest in probiotic clinical research among high-impact, quality journals. These data will help scholars choose journals for submission. Figure 5 shows Published in Molecular Biology/journal of Immunology and Medicine/Medical/Clinical journals are often published in Molecular Biology/Genetics/Health/Nursing/Medicine journal and Envirnmental/Toxicology/Nutrition/Molecular/Biology/Genetics/Health/Nursing/Medicine journals references. These findings indicate that current research on the clinical applications related to probiotics mainly focuses on basic research and transitional medicine. Most of the top ten references involve applications related to the nervous system, the gastrointestinal tract and metabolism. More and more evidence proves the microbiota not only a bidirectional pathway in the gut-brain axis, enabling the intestinal flora, including symbiotic bacteria, probiotics and pathogenic bacteria, to communicate through direct or indirect signaling pathways (35), activate neural pathways and the central nervous system (CNS) signaling system (36), but also directly interact with immune cells. It plays an important role in maintaining the immune balance of the gastrointestinal tract (37, 38), and plays a critical role in preventing and treating mental disorders, including anxiety, depression, and neurodevelopmental conditions (39–41). Meanwhile, Clostridium glitter, as a bile acid 7α-dehydroxylated intestinal bacterium, can also reduce diarrhea caused by intestinal infections (18). According to the highly explosive cited literature, the intensity and time intervals of the directions that researchers have been interested in in recent years can be referred to. From the 25 most frequently cited literatures, it can be seen that the most frequently cited literatures in recent years mainly involve the clinical applications of probiotics in gastrointestinal diarrhea, colitis, neurological diseases and specific diseases.

### 4.2 Research hotspots and frontiers

High-frequency keyword analysis reveals research hotpots and frontiers in this field. We employed keyword co-occurrence analysis to delineate the evolutionary trajectory of primary research foci and emerging trends in probiotic clinical applications (42). Cluster analysis of keywords combined with examination of the top 20 strongest citation bursts demonstrates both the broad clinical utility of probiotics and identifies current research hotpots in probiotic clinical applications. The main contents are as follows.

#### 4.2.1 Inflammation

The human body constitutes a complex holobiont, integrating human cells with symbiotic microbial communities. These microbiota colonize multiple anatomical sites, establishing dynamically balanced microecosystems that influence both local tissue homeostasis and systemic inflammatory regulation through various pathways (43), anti-inflammatory probiotics primarily exert systemic immunomodulatory and anti-inflammatory effects through indirect regulation of intestinal microbiota composition and function (44). Probiotic applications in inflammatory conditions have emerged as a predominant research focus in clinical practice over the past decade. Keyword hotspot analysis reveals their therapeutic potential across multiple inflammatory disorders, including diarrheal diseases, ulcerative colitis, Crohn's disease, necrotizing enterocolitis, atopic dermatitis, and periodontitis.

##### 4.2.1.1 Diarrhea

Intestinal dysbiosis, alternatively termed gut microbiota dysregulation, refers to the compositional and functional imbalance of microbial communities within the gastrointestinal tract (45). Current evidence suggests that gut dysbiosis serves as a primary etiological factor in the pathogenesis of numerous disease states (46, 47), Irritable bowel syndrome (IBS), one of the most prevalent functional gastrointestinal disorders, is clinically characterized by recurrent abdominal pain associated with altered bowel movement frequency and stool consistency (48). Indeed, both diarrhea and constipation result from: intestinal microbiota dysbiosis, compositional alterations in human gut microbiota, and dysregulation of complex microbiota-host immune system interactions (49). Probiotics, defined as live microorganisms administered as nutritional supplements, demonstrate significant potential in restoring gut homeostasis and ameliorating gastrointestinal symptoms (50, 51). Numerous clinical trials have demonstrated superior clinical efficacy of probiotic interventions compared to placebo controls, with multi-strain formulations outperforming single-strain preparations (52, 53). Radomańska et al. discovered five types of lactic acid bacteria (such as *Lactobacillus rhamnosus* LR110, *Lactobacillus paracasei* LPC100, *Lactobacillus acidophilus* LA120*, Lactobacillus casei* LC130, and *Lactobacillus plantarum* LP140) and four types of bifidobacterium (such as *Bifidobacterium breve* BB010 and *Bifidobacterium longum* BL020), *bifidobacterium bifidum* BF030, *Bifidobacterium lactis* BL040 and *Streptococcus thermophilus* ST250 can safely and effectively improve the overall symptoms of patients with IBS (54); Ishaque et al. also evaluated the effectiveness of 14 different types of probiotic mixtures (such as *Bacillus subtilis*, 4 types of Bifidobacteria, 7 types of lactic acid bacteria, *Lactococcus lactis* and *Streptococcus thermophilus*) against moderate to severe IBS (55); At the same time, the clinical trial by JanŁukasik et al. further confirmed that multi-strain probiotic formulations significantly prevent antibiotic-associated diarrhea (AAD), establishing their evidence-based utility in diarrheal prophylaxis (56). In conclusion, probiotics play a significant role in the occurrence and prevention of diarrhea, and this field is one of the hot applications.

##### 4.2.1.2 Ulcerative colitis

Ulcerative colitis (UC) is an intestinal inflammation, which caused by an imbalance between the intestinal microbiota and mucosal immunity (57). Patients with ulcerative colitis (UC) exhibit impaired functional diversity and ecosystem stability of gut microbiota, characterized by reduced Firmicutes abundance alongside increased Bacteroidetes and facultative anaerobes (58). Zhao-Hua Shen et al. performed a meta-analysis of 1,763 clinical cases, demonstrating that probiotic supplementation significantly enhances remission rates in active ulcerative colitis (UC) patients, especially *Lactobacillus* and *Escherichia coli* (59). In addition, adding lactic acid bacteria strains to conventional treatment can also reduce the recurrence rate of UC patients (60); Oliva et al. also demonstrated that topical administration of standard-dose *Lactobacillus reuteri* and *Escherichia coli* strains significantly reduces rectal mucosal inflammation in pediatric UC cases and promotes sustained clinical remission (61), co-administration of *Saccharomyces boulardii* with mesalazine therapy significantly alleviates clinical symptoms in patients with mild-to-moderate ulcerative colitis (UC) (62, 63). In a word, probiotics demonstrate dual therapeutic benefits for ulcerative colitis patients by both enhancing anti-inflammatory factor secretion and suppressing pathogenic gut bacteria through intestinal barrier and immune system modulation, as well as preventing and repairing pathogen-induced mucosal barrier dysfunction and associated epithelial damage.

##### 4.2.1.3 Atopic dermatitis

The skin is an important interface that constantly interacts with and perceives environmental stimuli, having evolutionarily adapted to maintain homeostatic equilibrium with its resident microbial communities comprising the cutaneous microbiome. With the development of sequencing technology, many studies have revealed the correlation between gut microbiota composition and human diseases, including allergic asthma and specific dermatitis (64, 65). The “gut-skin” axis has been scientifically established and is now recognized as a novel therapeutic target for both prevention and treatment of atopic dermatitis (AD) (66). The surface of the skin has a wide microenvironment with distinct physicochemical properties, necessitating selective colonization by microbiome species adapted to their specific anatomical niches (67). Current research demonstrates show that in areas rich in sebum, there are abundant lipid-dependent *Clostridium* and the fungus *Malassezia*, while in moist skin areas, more *Staphylococcus* and *Corynebacterium* are colonized (68). The lesion skin area of AD shows excessive growth of Staphylococcus aureus and expresses higher levels of virulence factors (69, 70). Notably, infants exhibiting reduced gut microbiota diversity demonstrate significantly higher susceptibility to AD compared to healthy controls, as evidenced by a cross-sectional study of 1,440 infant subjects (71); Enomoto et al. emphasized that perinatal supplement two types of bifidobacterium *(Bifidobacterium breve* and *Bifidobacterium longum*) before and after childbirth can reduce the risk of eczema and AD in infants (72); The meta-analysis conducted by Tan-Lim et al. indicates that the best probiotic formulations Mix8 (*Lactobacillus parabases, Bifidobacterium longum*) and Mix3 (*Lactobacillus rhamnoses, Bifidobacterium animalic*) exhibit the greatest efficacy in reducing AD risk (73). Probiotic supplements alter the intestinal environment, including regulating the composition of intestinal microbiota, preventing pathogen colonization, influencing bacterial metabolism, and restoring immune balance. These changes have all been proven to reduce inflammation in AD and improve clinical manifestations.

##### 4.2.1.4 Periodontitis

Periodontitis pathogenesis is primarily mediated by host immune responses that drive periodontal tissue destruction, with bacterial infection serving as the fundamental trigger for this immunopathologic cascade (74). Periodontal disease etiology primarily involves pathogenic bacterial colonization, commensal microbiota depletion, and host susceptibility. Patients with severe periodontitis may ingest 10^8^-10^10^ *Porphyromonas gingivalis* cells daily, a keystone periodontal pathogen (75). The therapeutic application of beneficial bacteria has emerged a novel approach for periodontal disease prevention and treatment. The therapeutic application of beneficial bacteria has emerged as a novel approach for periodontal disease prevention and treatment. The probiotic strains most frequently employed in periodontitis management include lactic acid bacteria, *Bifidobacterium*, and yeast. As highlighted in the review by Matsubara et al., oral probiotics demonstrate clinical utility as adjunctive therapy to scaling and root planning in periodontitis cases (76). Kuru et al. demonstrated that gingivitis patients pretreated with *Bifidobacterium lactis* exhibited significantly reduced IL-1β levels and elevated IL-10 concentrations in gingival crevicular fluid (GCF) compared to untreated controls (77); *In vitro* have further demonstrated the inhibitory effects of Bifidobacterium strains on periodontal pathogens, including *Porphyromonas gingivalis* (78). Oliveira et al. pointed that *Bifidobacterium lactis* can reduce the number of periodontal pathogenic bacteria on biofilms (79). While periodontitis exhibits multifactorial etiology, microbial dysbiosis persists as the primary pathogenic driver. Consequently, probiotics and their bioactive metabolites maintain demonstrable efficacy in promoting periodontal homeostasis.

##### 4.2.1.5 Bacterial vaginitis

Probiotics may help prevent female reproductive disorders by sustaining microbiome equilibrium. The vaginal microbiota balance is essential for optimal host-microbe interactions that maintain vaginal health (80), demonstrating therapeutic potential against bacterial vaginosis (BV), vulvovaginal candidiasis (VVC), sexually transmitted infections (STIs), trichomoniasis, human papillomavirus (HPV) infection, *Chlamydia trachomatis* infection, HIV susceptibility reduction, and genital herpes infection. BV represents the predominant cause of abnormal vaginal discharge in women aged 15–44 years, pathologically characterized by marked lactobacilli depletion accompanied by 100–1,000 fold proliferation of facultative and obligate anaerobes including *Gardnerella, Prevotella, Atopobium, Mobiluncus, Bifidobacterium, Sneathia, Leptotrichia* and BV-associated bacteria (81). Bastani confirmed *Lactobacillus* as a non-chemotherapy approach for restoring and maintaining normal urogenital flora, and indicated that probiotics, especially *Lactobacillus acidophilus, Lactobacillus rhamnosus* and *Lactobacillus fermentum*, administered in units for 2 months can maximize the normalization of vaginal flora, help cure existing infections and prevent the recurrence of BV (82); Ling et al. demonstrated that long-term probiotic administration demonstrates superior efficacy to conventional metronidazole therapy for bacterial vaginosis (BV). Their findings indicate probiotics stabilize the vaginal microbiome by reinforcing ecological homeostasis through suppression of pathogenic bacterial proliferation (83). Furthermore, current evidence indicates that although while oral probiotics reduce recurrent BV recurrence rates, intravaginal administration may provide more rapid therapeutic resolution (84, 85).

#### 4.2.2 Obesity

Obesity is defined as an abnormal or excessive fat accumulation that may be harmful to health (86). In recent years, it has been believed that changes in the bacterial strains residing in the human intestinal tract are pathogenic factors for obesity (87). Modifying the gut microbiota of obese individuals to enhance beneficial microorganisms or probiotics has been demonstrated as a therapeutic strategy against obesity and its associated disorders (88). More specifically, owing to their high pathogenicity barrier and antibiotic resistance, *Lactobacillus* and *Bifidobacteria* have been shown to reduce weight and fat accumulation to varying degrees (89). Cuffaro et al. pointed out that the abundance of specific bacterial species—including *Akkermansia muciniphila, Faecalibacterium prausnitzii, Eubacterium hallii, Anaerobutyricum hallii, Anaerobutyricum soehngenii, Bacteroides uniformis, Bacteroides coprocola, Parabacteroides distasonis, Parabacteroides goldsteinii, Hafnia alvei, Odoribacter laneus*, and *Christensenella minuta* is significantly decreased in obese individuals and patients with obesity-related metabolic disorders. This microbial depletion may contribute to impaired beneficial regulation of obesity. These metabolically active commensal microbes could serve as key players in obesity prevention and treatment strategies (90). Concurrently, certain probiotic-derived components and metabolites—including cell-free extracts, extracellular polysaccharides, and short-chain fatty acids—exert inhibitory effects on metabolic syndrome through modulation of multiple signaling pathways (91). Therefore, probiotic administration represents the most direct and effective clinical approach for managing obesity and obesity-related metabolic disorders, as it can selectively enrich beneficial microbial populations and ameliorate the gut microecological imbalance characteristic of obesity.

#### 4.2.3 Insulin resistance

Insulin resistance is characterized by either diminished pancreatic insulin secretion or impaired insulin sensitivity in target tissues, ultimately leading to elevated blood glucose levels (92). Numerous studies have shown a significant association between alterations in gut microbiota composition and diabetes pathogenesis (112). Notably, dysbiosis characterized by an altered *Bacteroidetes/Firmicutes* ratio correlates with enhanced intestinal permeability. The translocation of microbial metabolites across the compromised intestinal barrier can initiate downstream inflammatory cascades typical of diabetes. Importantly, specific bacterial taxa have been shown to confer protective effects through attenuation of pro-inflammatory mediators and preservation of intestinal barrier integrity, thereby mitigating diabetes risk. For example, *Lactobacillus fermentans, Lactobacillus plantarum, Lactobacillus casei, Rhodobacter, Achaemenophilus mucinophila* and *Bacteroides fragilis* (93). Asemi et al. have demonstrated that multi-species probiotic supplements (a mixture of *Lactobacillus* and *Bifidobacterium*) can reduce fasting blood glucose (FBG) levels in patients with insulin resistance (94); Tao et al. summarized 15 randomized controlled trials involving 902 individuals. The meta-analysis results further indicated that probiotic supplementation significantly reduces glycated hemoglobin (HbA1c) levels, fasting blood glucose (FBG), and insulin resistance in patients with impaired insulin sensitivity.” (95). Consequently, gut microbiota modulation through probiotic intervention may exert beneficial effects on diabetes control and its associated complications.

#### 4.2.4 Depression

It is well-established that the brain modulates gut function and shapes the gut microbial composition via autonomic nervous system regulation of intestinal transport, motility, secretion, and permeability (96). The gut microbiota, especially *Firmicutes* and *Bacteroidetes*, has been proven to influence mental health via the microbiota-gut-brain axis. Gut microbiota dysbiosis may be linked to mental disorders, including anxiety and depression. Currently, the treatment of depression is still mainly based on drug therapy. However, depression treatment remains primarily drug-based. However, orally administered pharmaceuticals substantially alter the gut microbiota. Studies indicate that clinically used antidepressants exhibit antibacterial properties, particularly against Gram-positive bacteria. Compared to drug-free controls, duloxetine increased the abundance of *Mycobacterium rectum* by more than 100 times (97). Evidence indicates that specific lactic acid bacteria strains (including *Lactobacillus plantarum, Lactobacillus paracasei, Lactobacillus rhamnosus* and *Lactobacillus brevis*) generate anti-inflammatory butyrates during metabolic processes. Elevated abundance of these strains may provide adjunctive benefits to antidepressant therapy by potentiating therapeutic effects (98). Wallace et al. administered *Lactobacillus* and *Bifidobacterium longum* supplements to 10 patients with major depressive disorder (MDD). Their results demonstrated that daily probiotic supplementation significantly alleviated anxiety symptoms (99). Consequently, probiotic-based interventions have emerged as a novel therapeutic approach for depression management.

#### 4.2.5 High blood lipid

Bile acids (BAs) are regarded as the key regulators of intestinal microbiota composition and function. Hepatic lipid metabolic efficiency determines the quantity of bile acids (BAs) entering the intestinal lumen, thereby influencing gut microbiota homeostasis (100), this dual regulatory mechanism inhibits bacterial overgrowth and preserves intestinal microbiota equilibrium, thereby mitigating intestinal barrier dysfunction (101). The clinical trial by Guerrero-Bonmatty et al. revealed that a triple-strain combination of Lactobacillus plantarum significantly reduced serum cholesterol levels in patients (102); Furthermore, a clinical trial by Park et al. involving 70 healthy adults showed that daily supplementation with *Lactobacillus plantarum* Q180 significantly improved postprandial lipid parameters indicators, including low-density lipoprotein cholesterol (LDL-C) and total cholesterol (TC), through gut microbiota modulation (103). These studies demonstrate that probiotics can modulate serum lipid profiles (TC, TG, HDL-C, and LDL-C) by enhancing probiotic colonization and regulating associated metabolites. Consequently, the involvement and mechanistic role of gut microbiota in hyperlipidemia pathogenesis have become increasingly evident. Microbiota-targeted therapeutic strategies for hyperlipidemia management and prevention represent a promising research frontier.

#### 4.2.6 Cancer

Through the gut-organ axis, the intestinal microbiota exerts both direct and indirect effects on immune responses and host physiology, thereby transforming cancer immunology (104). Specific cellular components of probiotic lactic acid bacteria may exert potential immunomodulatory effects, including regulation of cell-mediated immune responses, activation of the reticuloendothelial system, potentiation of cytokine signaling pathways, and modulation of interleukin and tumor necrosis factor production (105). Thirabunyanon et al. demonstrated that the viable probiotic strains of *Enterococcus faecium* and *Lactobacillus fermentum* in fermented milk effectively induced anti-proliferation effects in colon cancer cells *in vitro* (106); Toi et al. demonstrated in their study that regular intake of *Lactobacillus casei* probiotics combined with soy isoflavones significantly lowers breast cancer risk (107); Zhang et al. reported that *Lactobacillus acidophilus* and *Lactobacillus casei* produce bioactive compounds capable of inhibiting MCF-7 breast cancer cell proliferation (108). We observed that certain inhibitory compounds produced by probiotics exhibit beneficial effects against tumor cells. Escamilla et al. demonstrated that cell-free supernatants containing secreted bioactive macromolecules from *Lactobacillus casei* and *Lactobacillus rhamnosus* GG inhibited metastasis of human colon carcinoma cells (HCT-116) *in vitro* (109). Moreover, the probiotic *Acetobacter syzygii* exhibits prophylactic efficacy against oral cavity carcinoma, particularly squamous cell carcinoma. This demonstrates the beneficial role of probiotics in preventing oral diseases. Secretions from these strains demonstrate cytotoxic effects against oral cancer cell lines through apoptosis induction (110). In summary, as a valuable dietary supplement, probiotics contribute to reducing the risk of multiple cancer types while supporting the safety of conventional cancer treatments including chemotherapy, radiotherapy, and surgical procedures. The combination of probiotics with prebiotics demonstrates enhanced efficacy in both cancer prevention and treatment compared to probiotic use alone. Moreover, successful cancer prevention and treatment outcomes depend on specific probiotic strains, whether bacterial or fungal, along with appropriate dosage regimens and treatment duration.

## 5 Limitation

This study has several limitations. (1) To ensure high-quality bibliometric analysis, the investigation was restricted to English-language articles indexed in the Web of Science database. While Web of Science represents the most authoritative database for scientific publications across numerous research fields, this approach inevitably excluded studies published in non-SCI journals or other databases, introducing potential selection bias. Additionally, some high-quality publications might have been overlooked due to recent publication dates or low citation frequencies. Consequently, this bibliometric approach cannot fully substitute comprehensive systematic review methods. (2) The Cite Space and VOS viewer software packages present certain limitations. These analytical tools lack the capability to automatically consolidate keywords or subject terms with similar semantic meanings. Consequently, researchers must manually analyze and merge such terms, a process that may introduce potential inaccuracies in the research outcomes. (3) In identifying research hotspots and developmental trends, this study analyzed keyword frequency, centrality, and temporal distribution. While this approach is data-driven through literature keywords, it nevertheless retains an inherent degree of methodological subjectivity. (4) Bibliometrics analysis cannot evaluate the quality of individual studies as citation metrics are time-sensitive, More recent articles may receive fewer citations compared to earlier works primarily due to their publication date (111), These restrictions may have a slight impact on the overall results, but they do not affect the final results. Overall, our research provides a basis for understanding the research topics, hotspots and development trends of clinical applications related to probiotics.

## 6 Conclusion

At the dawn of the twentieth century, probiotics emerged as a distinct scientific concept. Over subsequent decades, probiotic-related publications increased exponentially. However, existing studies failed to provide a comprehensive overview encompassing literature distribution, research themes, and field frontiers, thereby limiting further advancement in this research domain. Building upon this foundation, the present study employs bibliometric visualization techniques utilizing VOS viewer and Cite Space software to analyze 3,674 probiotic-related clinical research articles indexed in the Web of Science core database. Through systematic keyword and core author selection, this investigation conducts a comprehensive, in-depth evaluation of publication patterns across temporal, geographic, institutional, authorial, and journal-specific dimensions, while innovatively assessing research themes within this field. Hot spots, collaborative networks and emerging trends collectively provide a macroscopic perspective on the current status and developmental trajectory of probiotic applications in medical research. This study reveals that scholarly attention in this field initially emerged in 2002. Our study offers foundational insights into current research within this field while identifying potential collaborators for researchers. The primary research foci currently center on probiotic applications in inflammatory conditions (including diarrhea, ulcerative colitis, dermatitis, periodontitis, and bacterial vaginosis), metabolic disorders (obesity, insulin resistance, and hyperlipidemia), mental health (depression), and oncology. Moreover, there has been a progressive increase in other clinical studies investigating probiotic applications. However, cooperation network among authors, institutions and countries within this research domain remain relatively fragmented, with only small clusters primarily concentrated in scientifically advanced developed and developing countries. These findings enable the academic community to identify emerging research directions and facilitate future clinical applications of probiotics, potentially serving as valuable references for subsequent investigations.

## Data Availability

The original contributions presented in the study are included in the article/supplementary material, further inquiries can be directed to the corresponding author/s.
